# Perceived Stress and Ocular Surface Symptoms: A Survey-Based Study of Medical Students

**DOI:** 10.3390/vision10040075

**Published:** 2026-10-04

**Authors:** Khaled Elubous, Nahla Alma’lawani, Khalid Abuelsamen, Aws Sbool, Karam Obiedat, Rama Al-Zahiri, Rand Ghazzawi, Osaid Al-Tamimi, Mohannad El-Faouri

**Affiliations:** 1 Department of Special Surgeries, Faculty of Medicine, The Hashemite University, P.O. Box 330127, Zarqa 13133, Jordan; muhannad_fa3ouri@hotmail.com; 2 Faculty of Medicine, The Hashemite University, P.O. Box 330127, Zarqa 13133, Jordan; nahlaalmalwani21@gmail.com (N.A.); khalidsamen2@gmail.com (K.A.); abuelsamen.03@gmail.com (A.S.); karamqobeidat@gmail.com (K.O.); ramaalzahiri@gmail.com (R.A.-Z.); randahmad018@gmail.com (R.G.); osaidt8@gmail.com (O.A.-T.)

**Keywords:** medical students, ocular surface disease index, perceived stress, PSS-10, symptomatic dry eye

## Abstract

**Background**: This study aimed to determine the prevalence of symptomatic dry eye among medical students and identify associated demographic and psychological factors. **Methods**: A cross-sectional online survey was conducted among medical students enrolled at Jordanian universities during September 2025. Participants completed a structured electronic questionnaire including demographic and lifestyle characteristics, the validated Perceived Stress Scale-10 (PSS-10), and the validated Ocular Surface Disease Index (OSDI). Univariable and multivariable logistic regression were used to identify factors associated with severe symptomatic dry eye. **Results**: A total of 311 medical students participated. The mean OSDI score was 34.84 ± 18.18, with 54.7% classified as having severe symptomatic dry eye. In multivariable logistic regression, perceived stress was associated with severe symptomatic dry eye after adjustment for measured covariates (adjusted OR = 1.056, 95% CI: 1.015–1.098, *p* = 0.007). Higher perceived stress was also associated with greater symptom severity (*p* = 0.001) and higher OSDI scores after adjustment (*p* = 0.010). **Conclusions**: Symptomatic dry eye symptoms were highly prevalent among medical students, and perceived stress was associated with greater symptom burden and severity after adjustment for measured covariates. These findings highlight the potential relevance of stress-related factors to ocular symptoms among medical students and encourage further investigation.

## 1. Introduction

Symptomatic dry eye is a common ocular surface condition characterized by ocular discomfort, dryness, burning sensation, foreign body sensation, fluctuating vision, and visual fatigue. Symptomatic dry eye can substantially impair visual performance, daily functioning, academic productivity, and quality of life, even in otherwise healthy young adults. The Tear Film and Ocular Surface Society Dry Eye Workshop II (TFOS DEWS II) defines dry eye disease as a multifactorial disease of the ocular surface characterized by loss of tear-film homeostasis [1,2].

Globally, estimates of dry eye prevalence vary substantially according to the population studied and the diagnostic criteria used. Prolonged digital-device use, reduced blink frequency, extended reading, inadequate sleep, and environmental exposures have been implicated as contributors to ocular surface symptoms [1,2,3,4].

Medical students may represent a particularly vulnerable population because their training involves prolonged reading, extensive digital screen use, irregular sleep schedules, sustained visual concentration, and substantial academic demands. Studies in young adults and medical students have reported a high frequency of dry eye symptoms, although prevalence estimates vary according to the assessment instrument and population [5,6].

Psychological stress has also emerged as a potential contributor to ocular surface symptoms. Proposed mechanisms include alterations in autonomic nervous system activity, tear secretion and homeostasis, inflammatory signaling, and the perception of ocular discomfort [6,7,8,9]. Stress may additionally influence behaviors such as screen exposure, sleep, and eye rubbing, which may further aggravate symptoms.

Therefore, the present study aimed to determine the prevalence of symptomatic dry eye among medical students using the validated OSDI and to evaluate demographic, lifestyle, and psychological factors associated with symptom presence and severity, with particular emphasis on perceived stress measured using the PSS-10.

## 2. Materials and Methods

### 2.1. Study Design and Population

This cross-sectional study was conducted using an online questionnaire developed through Google Forms. Data were collected from 24 to 30 September 2025. The target population comprised medical students aged 18–24 years enrolled at Jordanian universities, including Aqaba Medical Sciences University, Al-Balqa Applied University, the Hashemite University, Jordan University of Science and Technology, Mutah University, the University of Jordan, and Yarmouk University.

Participants were recruited using a non-probability convenience sampling approach through social media platforms. The inclusion criteria were active enrollment in medical school and age of 18–24 years. No specific exclusion criteria were applied.

Participation was voluntary. Electronic informed consent was obtained from all participants before questionnaire completion. Participants were informed of their right to withdraw at any time without consequence. Responses were anonymous, and confidentiality was maintained throughout the study.

### 2.2. Sample Size

The required sample size was calculated using the Epi Info™ StatCalc tool (version 7.2.5.0; Centers for Disease Control and Prevention, Atlanta, GA, USA). Assuming a statistical power of 80% and a significance level (α) of 0.05, the minimum required sample size was estimated to be 285 participants. A total of 414 survey submissions were received. Of these, 355 participants provided informed consent. Among the participants who consented, 350 provided sufficient responses to calculate an OSDI score. Following data cleaning, including exclusion of pilot/test submissions and patterned (“straight-line”) responses, 311 participants were included in the final analytic sample. This exceeded the prespecified minimum sample size of 285 participants.

### 2.3. Questionnaire and Measurements

The questionnaire was administered in English and consisted of three sections:

- Section 1: Demographic Characteristics and Potential Confounders

Participants provided information regarding age, sex, university, and academic year. Potential confounding variables included average sleep duration, daily screen time, and caffeine consumption.

- Section 2: Perceived Stress Assessment

Perceived stress was assessed using the Perceived Stress Scale-10 (PSS-10), a validated 10-item instrument measuring the degree to which individuals perceive situations in their lives as stressful and unpredictable [7]. PSS-10 scores range from 0 to 40 and were categorized descriptively as low (0–13), moderate (14–26), and high (27–40).

- Section 3: Dry Eye Symptoms

Dry eye symptoms were evaluated using the Ocular Surface Disease Index (OSDI), a validated 12-item questionnaire assessing ocular discomfort, visual function, and environmental triggers experienced during the preceding week [8]. An OSDI score ≥13 was used to identify symptomatic dry eye. Severity was classified as normal (0–12), mild (13–22), moderate (23–32), and severe (33–100), consistent with the classification used in the study and referenced to TFOS DEWS II [1].

### 2.4. Data Analysis

Data cleaning included exclusion of pilot responses (*n* = 3) and straight-lined responses (*n* = 36), defined as questionnaires in which participants selected the same response option across an entire matrix of questions without variation. These exclusions may have introduced potential selection bias. The electronic questionnaire required a response to each item and did not provide a “not applicable” option. Consequently, no OSDI items were unanswered among participants included in the final analytic dataset, and no imputation was required. OSDI scores were calculated using the standard fixed-denominator formula: (sum of the 12 item scores × 100)/48.

Descriptive statistics were used to summarize participant characteristics. Continuous variables were presented as mean ± standard deviation (SD), whereas categorical variables were presented as frequencies and percentages. Normality of continuous variables was assessed using the Shapiro–Wilk and Kolmogorov–Smirnov tests, in addition to visual inspection of histograms and Q–Q plots. Bivariate associations between continuous variables were examined using Spearman’s rank correlation coefficient (ρ), while categorical variables were compared using Pearson’s chi-square test.

Regression analyses were performed using the enter method. Univariable analyses were initially conducted to identify candidate variables for multivariable modeling. Variables with *p* < 0.25 in univariable analyses were considered for inclusion in the corresponding multivariable models. Binary logistic regression was used to identify factors associated with severe symptomatic dry eye, while ordinal logistic regression was used to assess factors associated with increasing symptom severity. For binary logistic regression, the outcome was dichotomized as severe symptomatic dry eye (OSDI score 33–100) versus non-severe symptoms (OSDI score 0–32). Participants with OSDI scores of 0–32, corresponding to normal, mild, or moderate symptoms, constituted the reference group. Multiple linear regression was used to evaluate the association between PSS-10 and OSDI scores after adjustment for variables meeting the prespecified inclusion criterion.

Categorical predictors were dummy coded using the indicator method, with the first or clinically appropriate category designated as the reference group. Model calibration for binary logistic regression was assessed using the Hosmer–Lemeshow goodness-of-fit test, while multicollinearity was evaluated using the variance inflation factor (VIF), with values <5 considered acceptable. All statistical analyses were performed using IBM SPSS Statistics version 25.0. A two-sided *p*-value <0.05 was considered statistically significant.

### 2.5. Ethical Considerations

The study protocol was reviewed and approved by the Institutional Review Board of the Hashemite University (IRB No. 7/5/2024/2025). The study was conducted in accordance with the ethical principles outlined in the latest revision of the Declaration of Helsinki.

## 3. Results

### 3.1. Demographic and Baseline Characteristics

A total of 311 medical students were included in the final analysis. The mean age was 22 ± 1 years, range of (18–24). Females represented 59.2% (*n* = 184) of the study population, while males represented 40.8% (*n* = 127). There were no missing data among participants included in the final analysis.

Regarding lifestyle characteristics, 57.6% (*n* = 179) reported more than 6 h of daily digital screen use. Most participants (55.6%, *n* = 173) reported an average sleep duration of 6–8 h per night, while 29.9% (*n* = 93) reported sleeping 4–6 h per night. Daily caffeine consumption was generally low to moderate: 50.2% (*n* = 156) consumed 1–2 cups of caffeine-containing beverages per day, whereas 32.2% (*n* = 100) reported no daily caffeine intake. The detailed demographic and baseline lifestyle characteristics are presented in Table 1.

### 3.2. Prevalence and Severity of Symptomatic Dry Eye

The mean OSDI score among study participants was 34.84 ± 18.18. According to the predefined OSDI severity categories, 54.7% (*n* = 170) were classified as having severe symptoms, 17.7% (*n* = 55) had mild symptoms, 16.4% (*n* = 51) had moderate symptoms, and 11.3% (*n* = 35) had normal OSDI scores.

### 3.3. Factors Associated with Severe Symptomatic Dry Eye

Univariable logistic regression analyses were initially performed for age, sex, average sleep duration, caffeine consumption, and PSS-10 score (Supplementary Table S1). In univariable analysis, PSS-10 score was significantly associated with severe symptomatic dry eye (OR = 1.051, 95% CI: 1.010–1.093, *p* = 0.015), whereas age, sex, average sleep duration, and caffeine consumption did not reach statistical significance.

A multivariable binary logistic regression analysis was conducted to identify independent factors associated with severe symptomatic dry eye. Age and sex were included a priori as standard demographic covariates, while average sleep duration and caffeine consumption were retained based on their potential relevance as behavioral factors and the prior literature. PSS-10 score met the prespecified univariable screening criterion of *p* < 0.25 and was included in the multivariable model. All five variables were entered simultaneously using the enter method. Multicollinearity was not identified, with VIF values of 1.05 for age, 1.05 for sex, 1.01 for sleep duration, 1.01 for caffeine consumption, and 1.01 for PSS-10 score. The Hosmer–Lemeshow goodness-of-fit test was non-significant (χ^2^ = 11.41, df = 8, *p* = 0.180), indicating no evidence of inadequate model fit.

Higher perceived stress was associated with increased odds of severe symptomatic dry eye after adjustment (OR = 1.056, 95% CI: 1.015–1.098, *p* = 0.007). Increasing age was associated with a slight reduction in the odds of severe symptoms, with each additional year associated with lower odds (OR = 0.958, 95% CI: 0.917–0.999, *p* = 0.047). Sex, average sleep duration, and caffeine consumption were not significantly associated with severe symptoms after adjustment. Table 2 presents the results for multivariable binary logistic regression analysis.

### 3.4. Association Between Perceived Stress and OSDI Scores

Simple linear regression demonstrated a significant positive association between perceived stress and OSDI scores (B = 0.486, SE = 0.176, *p* = 0.006), indicating that higher stress levels were associated with greater symptom burden. After adjustment for age and caffeine consumption, perceived stress remained significantly associated with higher OSDI scores (B = 0.455, SE = 0.176, *p* = 0.010).

### 3.5. Factors Associated with Symptomatic Dry Eye Severity

Ordinal logistic regression was performed to evaluate factors associated with progression across symptom severity categories (normal, mild, moderate, and severe). After adjustment for caffeine consumption, perceived stress remained significantly associated with increasing symptomatic dry eye severity (Estimate = 0.062, 95% CI: 0.025–0.100, *p* = 0.001).

## 4. Discussion

The present study demonstrated a high burden of symptomatic dry eye symptoms among medical students, with a mean OSDI score of 34.84 ± 18.18 and more than half of participants classified as having severe symptoms. These findings are consistent with the broader literature showing substantial variability in symptom prevalence among young adults and students, depending on population characteristics, diagnostic criteria, and assessment tool [5,6].

Importantly, this study identified perceived stress as an independent factor associated with severe symptomatic dry eye and demonstrated a positive adjusted association between stress and OSDI scores. Previous research has reported associations between psychological distress and dry eye symptoms, supporting the plausibility of a relationship between mental health and ocular symptom burden [9].

The association between stress and symptomatic dry eye is particularly relevant among medical students, who may experience substantial academic pressure, competitive environments, and irregular lifestyles. Previous research has documented considerable psychological distress among medical students [10,11].

Digital-device exposure is considered an important contributor to dry eye symptoms because prolonged screen use may reduce spontaneous blinking and increase incomplete blinking, contributing to tear-film instability [12]. In the present study, although 57.6% of participants reported more than six hours of daily screen time, screen time was not retained in the final multivariable model. This may reflect interactions with other behavioral or psychological factors, but the cross-sectional design prevents conclusions about mediation or causality.

Sleep duration has also been proposed as a contributor to dry eye symptoms. Although insufficient sleep was common in the present population, average sleep duration was not significantly associated with severe symptoms after adjustment. Previous studies have reported mixed findings, suggesting that sleep quality and related behavioral factors may be more informative than sleep duration alone [13].

Contrary to some previous reports, sex was not significantly associated with severe symptomatic dry eye in this cohort. Female sex is frequently reported as a risk factor for dry eye in population studies, although the strength of this association varies by age, diagnostic criteria, and population [2,14].

Caffeine consumption was not significantly associated with symptom severity in the present study. The relationship between caffeine and dry eye remains uncertain; a large population-based study found no significant association after adjustment for medical comorbidities [15].

While a high proportion of medical students in our study were classified in the severe OSDI category, this finding is broadly comparable with a recent meta-analysis of medical students, in which OSDI-based studies reported a pooled prevalence of 60%. Nevertheless, differences in outcome definitions and study methodologies should be considered when making comparisons [16]. The mechanisms underlying the relationship between psychological stress and ocular surface symptoms remain under investigation. This relationship may involve interconnected neuroendocrine, immune, and neurosensory pathways, including hypothalamic–pituitary–adrenal axis dysregulation, systemic and ocular inflammation, and oxidative stress. These proposed mechanisms provide biological plausibility for the association observed in our study; however, the cross-sectional design does not allow causal relationships to be established [17].

The study has several strengths, including a sample size exceeding the prespecified minimum, assessment of multiple potential predictors, and use of validated instruments for perceived stress and dry eye symptoms. The simultaneous assessment of ocular symptoms and psychological stress also provides clinically relevant information for a young medical-student population.

Several limitations should be acknowledged. First, the cross-sectional design limits the ability to establish temporal or causal relationships between perceived stress and symptomatic dry eye. Second, reliance on self-reported questionnaires may introduce response bias. Third, objective ophthalmological assessments, including tear break-up time, Schirmer testing, and ocular surface staining, were not available. In addition, convenience sampling through social media may limit generalizability beyond the participating student population and introduce self-selection and non-response bias. Moreover, we did not collect information on pre-existing mental health diagnoses, refractive status, the adequacy of vision correction or other environmental risk factors. These unmeasured factors may influence perceived stress and OSDI responses and therefore represent potential sources of residual confounding. Future longitudinal studies incorporating objective ocular-surface assessments are warranted.

## 5. Conclusions

Symptomatic dry eye symptoms were highly prevalent among medical students, with a substantial proportion reporting severe symptoms. Perceived stress was associated with both higher OSDI scores and greater symptom severity after adjustment for measured covariates. These findings highlight the potential relevance of stress-related factors to ocular symptoms among medical students and support further investigation of their relationship with ocular health. Longitudinal studies with objective ophthalmological assessments are needed to clarify the temporal and causal relationships between psychological stress and dry eye symptoms. The OSDI categories represent self-reported ocular surface symptoms and should not be interpreted as a clinical diagnosis of dry eye disease. The relatively high proportion of participants with severe symptoms may reflect the characteristics of the surveyed population, potential self-selection, and other unmeasured confounding factors. The relationship may also be bidirectional, as ocular discomfort may contribute to psychological stress.

## Figures and Tables

**Table 1:** Demographic and Baseline Characteristics of the Study Population (*n* = 311).

Variable	Category	*n*	%	Mean ± SD
Age (years)	—	—	—	22 ± 1
Gender	Female	184	59.2	—
	Male	127	40.8	—
Average sleep duration	<4 h	8	2.6	—
	4–6 h	93	29.9	—
	6–8 h	173	55.6	—
	>8 h	37	11.9	—
Average daily screen time	<2 h	5	1.6	—
	2–4 h	37	11.9	—
	4–6 h	90	28.9	—
	>6 h	179	57.6	—
Daily caffeine consumption	0 cups	100	32.2	—
	1–2 cups	156	50.2	—
	3–4 cups	48	15.4	—
	≥5 cups	7	2.3	—

**Table 2:** Multivariable Binary Logistic Regression Analysis for Severe Symptomatic Dry Eye.

Variable	OR	95% CI	*p* Value	VIF
Age	0.958	0.917–0.999	0.047	1.05
Female sex	0.971	0.604–1.562	0.904	1.05
PSS-10 score	1.056	1.015–1.098	0.007	1.01
Average sleep duration	0.908	0.631–1.307	0.604	1.01
Caffeine consumption				1.01
0 cups	Reference	—	—	
1–2 cups	1.273	0.763–2.125	0.356	
≥3 cups	1.375	0.694–2.724	0.361	

## Data Availability

The original contributions presented in the present study are included in the article. Further inquiries can be directed to the corresponding author.

## Supplementary Materials

The following supporting information can be downloaded at: https://www.mdpi.com/article/10.3390/vision10040075/s1. Supplementary Table S1. Univariable logistic regression analysis of factors associated with severe symptomatic dry eye.

## Abbreviations

OSDI	Ocular Surface Disease Index
PSS-10	Perceived Stress Scale-10
TFOS DEWS II	Tear Film and Ocular Surface Society Dry Eye Workshop II
IRB	Institutional Review Board
SD	Standard Deviation
OR	Odds Ratio
CI	Confidence Interval
VIF	Variance Inflation Factor
SPSS	Statistical Package for the Social Sciences
