# Supplementary material for: Perceived Stress and Ocular Surface Symptoms: A Survey-Based Study of Medical Students
Source: Vision (Basel). 2026 Oct 4;10(4):75. doi: 10.3390/vision10040075 (PMC13709878; doi:10.3390/vision10040075)
Supplement: Supplementary file 1 [file vision-10-00075-s001.zip › vision-4565176-supplementary.pdf]

Supplementary Table S1. Univariable logistic regression analysis of factors associated with severe symptomatic dry eye

Variable	OR	95% CI	p
Age	0.916	0.777–1.081	0.300
Female sex vs male	1.048	0.665–1.653	0.840
Sleep 4–6 h vs <4 h	2.568	0.579–11.392	0.215
Sleep 6–8 h vs <4 h	1.920	0.445–8.289	0.382
Sleep >8 h vs <4 h	1.759	0.366–8.455	0.481
Caffeine 1–2 vs 0	1.258	0.760–2.083	0.372
Caffeine ≥3 vs 0	1.471	0.756–2.862	0.256
PSS-10 score	1.051	1.010–1.093	**0.015**
